# Zurich's Contributions to 50 Years Development of Bruker

**DOI:** 10.1002/anie.201005067

**Published:** 2010-11-02

**Authors:** Richard R Ernst

**Affiliations:** * Laboratorium für Physikalische ChemieETH Zurich, Wolfgang Pauli Strasse 10, 8093 Zurich (Switzerland), Fax: (+41) 44 632 12 57

**Keywords:** analytical methods, history of science, NMR spectroscopy

Bruker Physik AG was founded in 1960 in Karlsruhe. However, the contributions originating from Zurich were also essential to the NMR development within the Bruker company. NMR spectroscopy proceeded like two red threads through the 50 year history of the company. As an “eye witness” (“Zeitzeuge”) I would like to follow the beginnings of the Zurich thread. Undoubtedly Bruker is today the undisputed world leader in nuclear magnetic resonance instrumentation, but beside NMR, all other areas of analytical spectroscopy have been equally competently and successfully covered. The limited length of this essay, unfortunately, does not permit us to adequately illuminate all relevant details, so as an eye witness I will concentrate more on my personal relationships with the company.

In 1955 Dr. Günther Laukien completed an impressive PhD thesis with the title “*Freie Präzessionen kernmagnetischer Momente*” (“*Free Precessions of Nuclear Magnetic Moments”*) at the Technical University of Stuttgart.[1] In 1958 his important Review article “*Kernmagnetische Hochfrequenzspektroskopie*” (“*High-Frequency Nuclear Magnetic Spectroscopy”*) was published in the *Handbuch der Physik*—*Encyclopedia of Physics*,[2] and only two years later, on September 7, 1960, the start signal for the founding of Bruker Physic AG was given. Günther Laukien was the main proponent of the company.[3]

**Figure 1:**
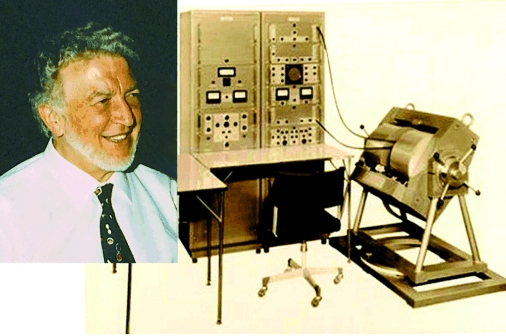
Professor Günther Laukien and his first pulse NMR spectrometer with a variable magnetic field—the predecessor to the B-KR322s pulse spectrometer, built in 1963 in Karlsruhe.

The initial production program of Bruker Physik AG was restricted to laboratory magnets and power supplies for use in physics. In light of the interests of Günther Laukien it is hardly surprising that from 1963 onward the company committed itself to nuclear magnetic resonance spectroscopy, which is today perhaps the most important scientific application of magnetic fields altogether.[4] Already in his PhD thesis Günther Laukien had dealt with high-frequency pulse methods for stimulating the free precessions of nuclear magnetic moments. This also became the program for “his” company, Bruker Physik AG, aiming at the investigation of materials in physics, chemistry, and material science. The production program filled a market niche with the first variable-frequency pulse spectrometer, B-KR322s (see Figure 1), and the company rapidly became financially viable.

The birth of Bruker Physik AG in 1960 should be seen against the background of the initial market leader in magnetic resonance spectroscopy, Varian Associates in Palo Alto, California. The Varian company was established twelve years earlier on April 20, 1948, within the Stanford Industrial Park by scientists from Stanford University.[5–7] They intended to commercialize two fundamental patents: the invention of the klystron in the year 1937 by Russel Varian and the (co)discovery of nuclear magnetic resonance spectroscopy by Felix Bloch in 1946. Both inventions took place at Stanford University and, as is known, Edward M. Purcell and Felix Bloch were honored with the Nobel Prize for Physics in 1952 for the discovery of nuclear magnetic resonance spectroscopy.

While the klystron found direct application as a microwave amplifier in physics and radar technology, it was recognized early on at Varian that NMR had an almost unlimited range of applications in chemistry. This led to the development of routine instruments for chemical structural analysis (and later also in molecular biology). The most successful Varian instruments, such as the HR30 (1952), HR40 (1955), HR60 (1958), and HR100 (1959), were all high-resolution solution-state spectrometers that were based on continuous-wave sweep methods and on electromagnets. The real breakthrough in the practical application of NMR was achieved with the introduction of the legendary A-60 spectrometer in the year 1961.[8, 9] It reached a “fame” comparable to that of the Ford Model T among the early automobiles. The A-60 was easy to operate and proved to be enormously useful in practical routine work.

Initially Bruker Physik did not have an equivalent to compete with the A-60, and their earliest NMR spectrometers mainly found application in physical research laboratories. Access to routine applications in chemistry remained barred to Bruker at the beginning. The expansion toward high-resolution NMR in solution came from a different side.

In 1952 Hans Heinrich Günthard was appointed as the professor of physical chemistry at the ETH Zurich. He realized that European chemistry research in the 1950s had, in contrast to research in America, great deficiencies in the application of modern physical chemistry, particularly of spectroscopic analysis methods. At that time, physical chemistry at the ETH in Zurich had dulled to the point of parody and was content to explore classical thermodynamics. This was in stark contrast to the organic chemistry flourishing at the ETH under professors and Nobel Laureates Leopold Ruzicka and Vladimir Prelog. Both recognized the deficits and supported the youthful initiatives of Günthard with word and deed. Subsequently, in admiration of American role models, Günthard built modern spectrometers with his co-workers for almost all frequency ranges including infrared and microwave spectrometers, electron spin resonance and nuclear magnetic resonance spectrometers.[10] Günthard became institute director and professor of physical chemistry in 1959 as the successor of Gottfried Trümpler, and he radically “decluttered” the laboratory for physical chemistry.

Hans Heinrich Günthard, came indirectly to academia and had a special affinity for those with unusual careers. He found in Hans Primas a congenial colleague. Hans Primas came without a formal education and with “only” a chemistry diploma from the Technikum of Winterthur to the Günthard group. From 1953 he was occupied with the theory and practice of infrared spectroscopy.[11–14] Günthard was impressed with his performance and entrusted him with an even more difficult project: the construction of an entire nuclear magnetic resonance spectrometer, including all the components—magnet, electronic console, and probe head. As a practical chemist Primas did not have much knowledge of electronics, and in the shortest time learnt the basics through to the point of application of most modern components, such as, the “nuvistor”, a form of miniature electronic tube with metal housing. Primas also learnt, with lighting speed, linear response theory dealing with frequency and pulse response, as well as feedback and stability theory. In the circles of Günthard “Learning by Doing!” and “Trial and Error!” were daily practice. Nothing was impossible from the outset. And in the hands of Hans Primas everything appeared to work.

**Figure 2:**
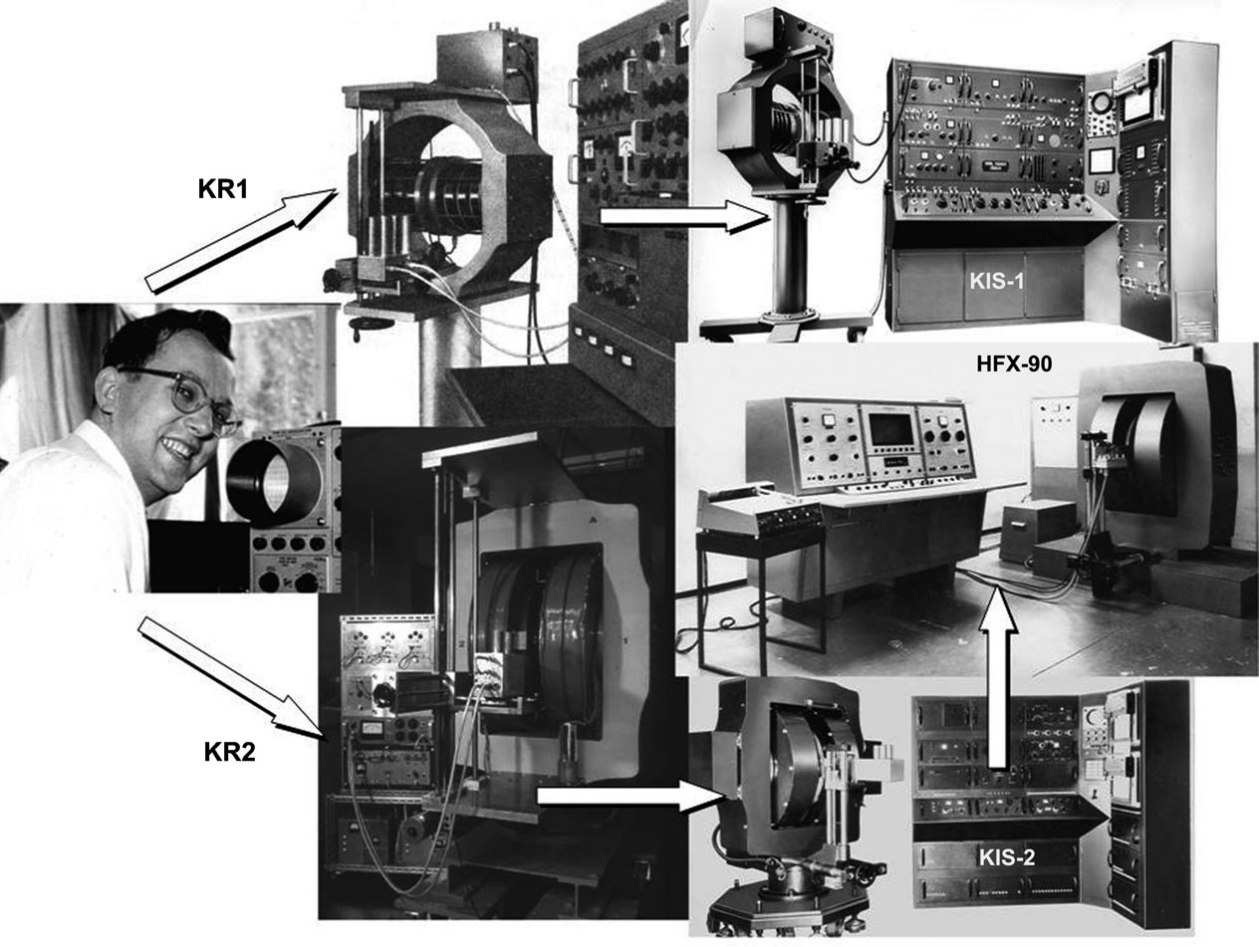
Development of the high-resolution NMR spectrometers based on the pioneering work of Hans Primas at the ETH Zurich with the KR-1 (25 MHz proton resonance frequency, 1957) and the KR-2 (75 MHz, 1961). These led to the subsequently designed spectrometers KIS-1 (25 MHz) and KIS-2 (90 MHz, 1963) from Trüb, Täuber Co. AG, and ultimately to the Bruker–Spectrospin spectrometer, HFX90 (90 MHz), built in 1967.

Between the years 1954–1958 Hans Primas developed a novel NMR spectrometer.[15–17] The commercial Varian spectrometers were world class at that time and served as archetypes. However, out of pride and thriftiness Günthard did not want to buy one from Varian. In contrast to the Varian instruments, the first Zurich NMR spectrometer, KR1, used a permanent magnet, and was, thus, restricted to a low proton resonance frequency of only 25 mhz (see Figure 2). Numerous innovations were simultaneously implemented, such as spherical NMR sample tubes and a powerful field stabilizer.[18] On April 28, 1955 the first ethanol spectrum was observed. In the course of 1957, routine proton resonance spectra of acceptable quality could be measured.[19] The limited performance of the spectrometer with 25 mhz proton resonance, particularly its long measurement times owing to the low frequency, hindered the its useful employment in chemistry. Nevertheless, several application spectra were measured.[20, 21] Particularly Prof. Vladimir Prelog was interested in applications in organic chemistry.

From 1958 to 1962, during his PhD studies, the author assisted Primas in his electronic NMR developmental work.[22–26] There is no question that this “apprenticeship” under the guidance of “Master Primas” was of major significance for the author′s later success in NMR instrumentation and methodological development, leading to Fourier transform spectroscopy and to multidimensional NMR!

Already, in the year 1955, Günthard arrived at the courageous decision to fertilize the Swiss industry with the nascent NMR spectrometer, KR1. Thus, in 1955 negotiations were initiated with the Zurich-based company Trüb, Täuber & Co. AG for the commercial reproduction of the KR1. At that time Trüb, Täuber & Co. AG was a traditional electrical engineering company that produced mainly display instruments, such as volt- and ampere meters. On the side, since 1929, they had operated a department for scientific instruments under Dr. Lieni Wegmann, in which electron microscopes and high-voltage cathode-ray oscilloscopes had been developed. The production of NMR spectrometers seemed daring, but not impossible. The new instrument KIS-1[27] was advertized as “European excellence in the field of nuclear physics technology”[28] (“Eine europäische Spitzenleistung auf dem Gebiete der kernphysikalischen Technik”). The success was respectable but not overwhelming (see Figure 2). Some 12–15 KIS-1 spectrometers found admission to university research laboratories in central Europe.

Simultaneously, a higher field 90 MHz spectrometer, KR2, was developed by Prof. Hans Primas and his group at the ETH Zurich. Ultimately, however, the spectrometer only managed a proton resonance frequency of 75 MHz because of the limitations of the self-constructed electromagnet. On the other hand, specially shaped, innovative pole pieces (“Primas-Polschuhe”) optimized the homogeneity of the field independently of its strength by avoiding local saturation effects.[29] An NMR field frequency stabilizer was also developed. Thus, a modern prototype spectrometer emerged. This instrument was also reproduced by Trüb–Täuber and was brought to the market in 1963 under the name KIS-2 as a 90 mhz spectrometer (see Figure 2).[30] Only a few instruments of this type found their way into practical applications, for example, at the SHELL Basic Research Institute at Birlinghaven.[31]

In the meantime, the financial problems of Trüb–Täuber grew also in sections other than the NMR department, and in 1965 the company closed. In this situation Günther Laukien announced his interest in the acquisition of the NMR department. This took place on June 24, 1965, and the company Spectrospin was founded.[32] As with all takeovers, besides promising products—in this case the prototype spectrometer, KIS-2—the gain of highly valued employees was crucial, namely Dr. Werner Tschopp and (Dr.) Tony Keller. Both left a lasting influence on the young company, Spectrospin. Tony Keller has in the meantime been awarded three honorary doctorates (TU Berlin, University of Queensland, and University of Florence). The first milestone of the young firm was set in 1967 by the first complete transistorization of an NMR spectrometer, the HXF-90[33] (see Figure 2).

The extension of the product line to high-resolution NMR was essential for the later success of Bruker. Two years after the foundation of Spectrospin AG, Bruker was still hopelessly behind the market leader Varian. In 1967, of the 131 NMR spectrometers installed in Germany 122 were from Varian, six from Bruker, and three from Trüb–Täuber.[34] There was an enormous effort required to close the gap in the number of installed instruments. For this, innovative technology was required, much more than merely clever sales tricks.

I experienced only from a distance the foundation and early years of Spectrospin as a department of Bruker Physik Co. AG. From 1963 to 1968 the author was employed at the rival company Varian Associates in Palo Alto. In fact, this was the author′s obligatory postdoctorate in the USA in the form of an employment in industry. It was his personal wish to avoid the ivory-tower atmosphere of academia, by an industrially and socially relevant occupation, and he wished never to return to a university. This helped to compensate for his lack of self confidence as a “useless academic”. In hindsight, the highly relevant research work at Varian Associates fulfilled this aim optimally. After all, it led to the Nobel Prize for chemistry in the year 1991. But the prize-worthy “invention” within the competitive Varian Company turned out to be also highly significant for the later development at Bruker–Spectrospin.

The crucial idea for the improvement in sensitivity of NMR by parallel data acquisition originated from the author′s ingenious boss at Varian, Weston A. Anderson. Its first implementation by pulse stimulation and by data analysis through Fourier transformation was implemented by the author. The resulting NMR–Fourier spectroscopy method was patented and published.[35–37] It later revolutionized practical NMR spectroscopy. However, the leaders at the time at Varian did not recognize its significance and missed a unique commercial opportunity, probably because the original founders of the company with a sound scientific background were, in the meantime, replaced by profit oriented but technically illiterate MBAs and lawyers. At this point the decline of the market leader, Varian Associates, slowly began to take its course.

Astonishingly, the first commercial implementation of the Fourier concept took place at Bruker, and in 1969, through the initiatives of the innovative Tony Keller, it was demonstrated at the *Pacific Conference on Chemistry and Spectroscopy* at Anaheim, California, in October 1969, with an intriguing application to ^13^C resonance. This led to the “only-Fourier” NMR spectrometer, WH90, in 1972 (the notation WH for workhorse being quite appropriate!).[38] A sweep supplement had been planned and offered, but never produced for this spectrometer. This underlines the striking success of the Fourier method in NMR spectroscopy.

The author′s return to Switzerland and to the ETH Zurich in 1968 was rather unpleasant: “the prophet has no honor in his own country”. He did not receive adequate instrumental and personnel resources, and one year later he suffered a nervous breakdown, from which he recovered only very slowly. Alongside his teaching activities the author still acted as a consultant for Varian. In research he developed advanced methods in Fourier spectroscopy, which were usually patented by Varian in Palo Alto.

In 1971, at an AMPERE Summer School in Yugoslavia the inspiration for the development of two-dimensional NMR spectroscopy came from the Belgian physicist Jean Jeener. This gave the research group a new impetus and also led to the conception of medicinal Fourier imaging methods. A later very lucrative patent[39] for this was also left to Varian for a “tip” of 200 US$. However, over time, the distance to California became too large for an efficient collaboration and the author welcomed the invitation from Günther Laukien to transfer consultation activities from Palo Alto to Fällanden and Bruker–Spectrospin. This was also rather fitting for the author′s patriotic sense of responsibility.

**Figure 3:**
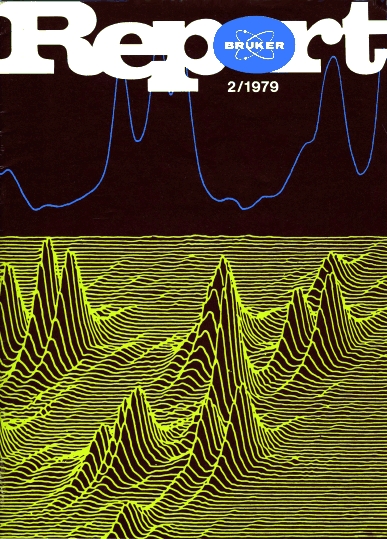
2D *J*-Resolved spectroscopy. The cover picture of the Bruker Report (2/1979) illustrates a paper published therein by Dr. Enrico Bartholdi, an employee of Bruker–Spectrospin and a former graduate student of R. R. Ernst. A 2D *J*-resolved proton resonance spectrum of the cyclic peptide AW 27–400 (*M*_W_ 1201) is shown with the methyl group signals in the region of *δ*=0.8–1.1 ppm resolved along the second dimension with the *J*-splitting in the region of ±10 Hz.[40]

The intensive cooperation with Bruker–Spectrospin led primarily to the development of multidimensional NMR spectroscopy.[40] In this area, Bruker was once again ahead of the competition. In addition to high-field spectroscopy, this area was of great significance for the structure determination of biological macromolecules in solution. The early, intense collaboration with the research groups of R. R. Ernst[41, 42] (see Figure 3) and K. Wüthrich[43, 44] at the ETH in Zurich earned the company pivotal advantages. Numerous patents arose from this triple cooperation (e.g., Refs. [45] and [46]). At this time Varian also had a similar fruitful collaboration with the group of R. Freeman in Cambridge. However, the two Nobel Prizes in chemistry in 1991 and 2002 indirectly gave Bruker a unique shine.

The highly successful development of superconducting high-field spectrometers became an additional milestone for Bruker,[47, 48] with the worldwide first 1000 mhz proton resonance instrument.[49] Varian had in fact pioneered this area, but then regrettably gave up the development of superconducting magnets in the 1970s, being misjudged as irrelevant.[50] Superconductors turned out also to be of great relevance for the design of cryoprobes for the improvement of sensitivity by up to a factor of ten.[51–54]

In light of these achievements and successes it is not surprising that today Bruker has left its rivals far behind in the NMR market share. Exact figures are difficult to determine, however, it is said that the current worldwide lead of Bruker in NMR spectroscopy lies at about 80 %, and thus without doubt Bruker has become the clear market leader. This development is remarkable, when one compares it to the year 1967, when even in its homeland Germany the market share of Bruker, including Trüb–Täuber, at the most lay at 7 %,[34] and worldwide it was even lower!

NMR spectroscopy dominated the pioneering phase of the company, and is still today the flagship of Bruker Corporation. But present-day activities go far beyond NMR: magnetic resonance imaging (MRI)[55] has become very important. Electron paramagnetic resonance (EPR) was already dealt with early on.[56, 57] And when in 1975 Varian abandoned EPR completely for reasons that are hard to understand,[58] Bruker became the undisputed market leader also in EPR. Bruker Daltonics encompasses today a broad class of ion beam analysis methods,[59] and Bruker X-ray technology supplies indispensable analytic methods.[60]

**Figure 4:**
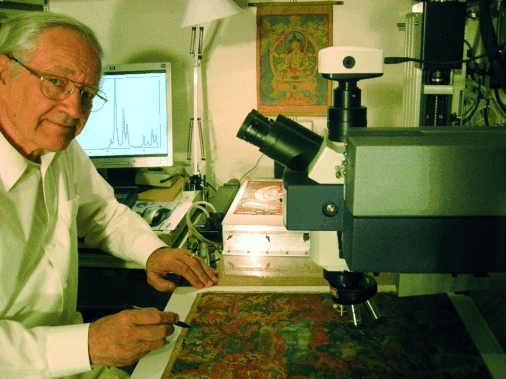
A Bruker Optics SENTERRA Raman microscope in an optimized configuration and environment, arranged for the nondestructive identification of pigments in large Tibetan scroll paintings in the private laboratory of the author in Winterthur. The screen shows the Raman spectrum of orpiment (As_4_S_6_).

For this contemporary witness, Bruker Optics[61] has recently gained special significance. He has today at his disposal an advanced SENTERRA Raman spectrometer from Bruker Optics[62] in his private home for his “spare-time passion”. With it, he is today able to perform highly enlightening nondamaging pigment analysis of Tibetan and Central Asian scroll paintings. In this way the author is able to obtain valuable insightful information about age, provenance, and painting techniques in Tibetan cultural treasures.[63, 64] The eye witness comes full circle with this quite unexpected application of a Bruker spectrometer. It links his cultural historic and his spectroscopic interests in a perfect match (see Figure 4).

And what is the moral of the success story of the Bruker Corporation? Bruker has been active in a unique manner in a high-tech leading field over an incredibly long period of 50 years. Few other companies can match Bruker. Whatever Bruker has achieved has demanded novel technologies that in each case would have been impossible a few years earlier. For academia, such companies are ideal partners: they are highly ambitious, stimulating, and grateful for creative contributions. This form of exchange can indeed be highly motivating for inventive entrepreneurs and for innovative researchers with endurance. Ultimately, it is courageous and creative efforts that are decisive. The example of Varian also shows that initial success can lead to complacency and the underestimation of motivated competitors. One further lesson that can be drawn concerns company leadership. One should guard oneself from companies that are managed by technically illiterate lawyers and MBAs. Technical expertise coupled to close contact with the market remains essential for avoiding flawed decisions. Bruker has in the past evaded such perils through wise personnel policies and long-term continuity. The company has produced beneficial innovations and added value for 50 years. Also investors might derive lessons: it is still the rock-solid technical expertise of a CEO together with their market awareness that offer the best guarantees against false decisions. With innovative companies, like the Bruker Corporation, the frequently forecast demise of Europe is not imminent. Even if the Bruker Corporation is of late governed by American law, the spirit of this remarkable company is still firmly European.
